# Feeding Issues in Infants Referred for Frenotomy

**DOI:** 10.7759/cureus.59539

**Published:** 2024-05-02

**Authors:** Mattie Rosi-Schumacher, Alison C Ma, Alyssa Reese, Ryan Nagy, Jason C DeGiovanni, Mark Nagy, Michele M Carr

**Affiliations:** 1 Otolaryngology, University at Buffalo Jacobs School of Medicine and Biomedical Sciences, Buffalo, USA

**Keywords:** frenuloplasty, frenulectomy, lingual frenotomy, tongue tie, ankyloglossia

## Abstract

Introduction: The diagnosis of ankyloglossia has increased significantly around the world over the last decade. Frenotomy is indicated in infants with ankyloglossia to improve breastfeeding, although there is little scientific evidence of its efficacy. The purpose of this study is to evaluate whether infants being referred for frenotomy had feeding issues prior to the procedure.

Methods: A retrospective chart review was undertaken for all infants under one year of age referred with ankyloglossia to a pediatric otolaryngology practice or a pediatric hospital between 2018 and 2020. Data included age at referral, gender, comorbidities, feeding issues, whether ankyloglossia was diagnosed, and whether frenotomy was done. Frequencies and non-parametric comparisons were calculated.

Results: Of the 646 consultations made for tongue tie, a diagnosis of ankyloglossia was made in 94.7% (N=612) of the patients based on clinical judgment. The most common feeding complaints were poor latch (57.1%, N=369) and painful latch (50.3%, N=325). Eighty one (12.5%) patients did not have a reported feeding difficulty. Most patients had an anterior tongue tie (85.8%, N=554), with some showing signs of restricted tongue movement (30.1%, N=184). Ankyloglossia was 4.03 times more likely to be diagnosed (p<.001) and frenotomy was 1.76 times more likely to be performed (p<.001) in the hospital setting compared to the clinic setting.

Conclusion: Children under the age of one referred to otolaryngology for ankyloglossia were often diagnosed concordantly, although some lacked feeding issues that would indicate frenotomy. There are still knowledge gaps about infantile ankyloglossia in referring medical personnel.

## Introduction

Ankyloglossia, more commonly known as tongue tie, refers to an abnormal, short lingual frenulum that is associated with restricted tongue movement. While the prevalence of this congenital anomaly is not well understood, a recent systematic review found that the overall prevalence was 8% in children younger than one year of age. Ankyloglossia has been associated with breastfeeding difficulties and speech disturbances. If treatment is deemed necessary, infants may undergo an incisional release (frenotomy), excision of the frenulum (frenectomy), or repositioning of the frenulum tissue (frenuloplasty) in a clinic, newborn nursery, or operating room [1]. 

Over the past decade, an increase in ankyloglossia diagnosis has been noted [2]. The most common explanations center around the increased focus on breastfeeding and the widening definition of ankyloglossia due to the lack of good quality conclusive research in the area. The American Academy of Otolaryngology-Head and Neck Surgery Foundation (AAO-HNS) reported expert consensus that ankyloglossia was overdiagnosed in some communities and a significant number of children have received unnecessary lingual frenulum surgery. Specifically, not all infants with breastfeeding difficulties will have ankyloglossia, and not all infants with ankyloglossia require surgical correction [3]. Furthermore, early frenotomy in infants less than one-month-old is generally agreed to be the most beneficial, but there is no agreed-upon preferred location for frenotomy, in a clinic or under general anesthesia [3]. Thus, to better understand the increasing incidence of ankyloglossia and the over-diagnosis of the condition, this study sought to understand whether ankyloglossia referrals are made appropriately to pediatric otolaryngologists based on eventual diagnosis and feeding complaints.

## Materials and methods

This study was approved by the University at Buffalo Institutional Review Board (Study#00004967). All children referred for frenotomy under one year of age at John R. Oishei Children’s Hospital and UBMD pediatric otolaryngology clinic between January 2018 and December 2020 were included in the study. A list of inpatients was generated using billing records for otolaryngology consults with an ICD-10 code for ankyloglossia (Q38.1). Outpatient clinic charts were found by going through day sheets which listed the chief complaint; if tongue tie was listed, the chart was reviewed, and the patient was included if the visit was in fact for evaluation of ankyloglossia.

From patient charts, demographic information regarding the infant’s sex, weight on the day of evaluation, gestational age, and co-morbidities was recorded. Feeding complaints reported by mothers were noted as either painful latch, poor latch, prolonged feeding, poor weight gain, or other. Any descriptions of the tongue and oral tissue were also noted. Lingual frenulum descriptors such as notched tip, taut frenulum, and lack of extension beyond the alveolar ridge were specifically noted. Diagnoses of ankyloglossia were made by each provider according to their personal method. Lastly, whether a diagnosis of ankyloglossia was made and whether frenotomy was performed were recorded. Chart review was performed by two authors (AM and MC). Frequencies and non-parametric comparisons were analyzed with IBM SPSS Statistics for Windows, Version 27 (Released 2020; IBM Corp., Armonk, New York, United States) and statistical significance was set at p<.05.

## Results

Demographics

A total of 646 infants under the age of one were included in the study. Table 1 shows the distribution of patients by location and provider. Just over half of the consultations took place in the inpatient setting (N=359, 55.6%). The mean age of patients was 28.2 days (95% CI 23.5-33.0). Four hundred and six (62.8%) patients were male, 236 (36.5%) were female. Sex was not reported for four patients. Outpatients referred for ankyloglossia were significantly older, with a mean age of 54.8 days (95% CI 45.5-64.0), compared to 7.0 days (95% CI 4.2-9.8) for inpatients (p< .001). Sex was not significantly different between locations (Table 2).

**Table 1:** Consultation locations and providers

Location		N (%)
Clinic		287 (44.4)
	Pediatric Otolaryngologist	287 (100)
Hospital		359 (55.6)
	Pediatric Otolaryngologist	206 (57.4)
	Otolaryngology Resident Supervised by Attending	144 (40.1)
	Pediatric Otolaryngology Nurse Practitioner	9 (2.5)

**Table 2:** Demographics of the study group Age p-value calculated using Spearman’s Rank Correlation.  Gender p-values calculated using the chi-square test.  Age converted to days from months using (365/12)*age in months.

	Total	Outpatient	Inpatient	p-Value
Age (days), mean (95% CI)	28.2 (23.5-33.0)	54.8 (45.5-64.0)	7.0 (4.2-9.8)	< .001
Sex, N (%)				
Female	236 (36.5)	98 (34.1)	138 (38.4)	.320
Male	406 (62.8)	185 (64.5)	221 (61.6)	-

Diagnosis

Of the 646 consultations made for tongue tie, a diagnosis of ankyloglossia was made in 612 (94.7%) patients. Most patients referred for ankyloglossia were found to have an anterior tongue tie (N=554, 85.8%). Posterior ankyloglossia was significantly more likely to be diagnosed in the inpatient referrals (p=.007) and no ankyloglossia diagnosis was more likely to be made in the outpatient referrals (p<.001). Anterior ankyloglossia diagnosis was not significantly different in the hospital or clinic setting (p=.799) (Table 3). 

**Table 3:** Ankyloglossia diagnosis p-values calculated using the chi-square test

Ankyloglossia Diagnosis, N (%)	Total	Outpatient	Inpatient	p-Value
None	34 (5.3)	26 (9.1)	8 (2.2)	< .001
Anterior	554 (85.8)	245 (85.4)	309 (86.1)	.799
Posterior	58 (9.0)	16 (5.6)	42 (11.7)	.007

Feeding parameters

In both the inpatient and outpatient settings, painful latch (N=325, 50.3%) and poor latch (N=369, 57.1%) were the most common complaints seen. Painful latch (p=.004) and poor latch (p<.001) were significantly associated with a diagnosis of ankyloglossia, while prolonged feeding (p=.124), poor weight gain (p=.396), and other feeding difficulties (p=.051) showed no significant association. When a diagnosis of ankyloglossia was made, painful latch (p<.001) and poor latch (p<.001) were more likely to be seen in the inpatient setting, while prolonged feeding (p=.014), poor weight gain (p<.001), other feeding complaints (p=.003), and no feeding complaints (p<.001) were more likely to be seen in clinic. There were no significant differences in feeding complaints when ankyloglossia was not diagnosed. Unsurprisingly, infants with no feeding difficulties were more likely to ultimately not be diagnosed with ankyloglossia (p=.002) (Table 4).

**Table 4:** Breastfeeding complaints p-values calculated using the chi-square test

Feeding Complaints, N (%)	Ankyloglossia Diagnosis				No Ankyloglossia Diagnosis				p-Value for Comparison of Ankyloglossia vs No Ankyloglossia
	Total (N=612)	Clinic (N=261)	Hospital (N=351)	P Value	Total (N=34)	Clinic (N=26)	Hospital (N=8)	p-Value	
Painful Latch	316 (51.6)	83 (31.8)	233 (66.4)	< .001	9 (26.5)	8 (30.8)	1 (12.5)	.306	.004
Poor Latch	360 (58.8)	106 (40.6)	254 (72.4)	< .001	9 (26.5)	7 (26.9)	2 (25.0)	.400	< .001
Prolonged Feeding	22 (3.6)	15 (5.7)	7 (2.0)	.014	3 (8.8)	3 (11.5)	0 (0.0)	.314	.124
Poor Weight Gain	33 (5.4)	26 (10.0)	7 (2.0)	< .001	3 (8.8)	2 (7.7)	1 (12.5)	.675	.396
Other	87 (14.2)	50 (19.2)	37 (10.5)	.003	9 (26.5)	7 (26.9)	2 (25.0)	.914	.051
None	71 (11.6)	49 (18.8)	22 (6.3)	< .001	10 (29.4)	7 (26.9)	3 (37.5)	.566	.002

Tongue description

Restricted tongue movement was the most common tongue descriptor used to describe patients diagnosed with ankyloglossia (N=184, 30.1%). Notched tip (p<.001), no extension beyond the alveolar ridge (p<.001), taut frenulum (p=.038), short lingual frenulum (p<.001), and restricted tongue movement (p<.001) were significantly associated with those diagnosed with ankyloglossia. Inpatients diagnosed with ankyloglossia were significantly more likely to be described as having restricted tongue movement (p<.001), while outpatients were more likely to have a short lingual frenulum (p<.001), a taut frenulum (p=.022), and limited extension beyond the alveolar ridge (p<.001) (Table 5). No significant association for any tongue descriptor was found for patients who were not diagnosed with ankyloglossia regarding clinic versus hospital setting.

**Table 5:** Tongue descriptors p-values calculated using the chi-square test

Tongue Descriptions, N (%)	Ankyloglossia Diagnosis				No Ankyloglossia Diagnosis				Total p-Value
	Total (N=612)	Clinic (N=261)	Hospital (N=351)	P value	Total (N=34)	Clinic (N=26)	Hospital (N=8)	P Value	
Normal	9 (1.5)	8 (3.1)	1 (0.3)	.080	33 (97.1)	25 (96.2)	7 (87.5)	.428	< .001
Notched Tip	79 (12.9)	58 (22.2)	22 (6.3)	.766	0 (0.0)	0 (0.0)	0 (0.0)	-	< .001
No Extension Beyond Alveolar Ridge	76 (12.4)	35 (13.4)	41 (11.7)	< .001	0 (0.0)	0 (0.0)	0 (0.0)	-	< .001
Taut Frenulum	38 (6.2)	22 (8.4)	16 (4.6)	.022	1 (2.9)	1 (3.8)	0 (0.0)	.581	.038
Short Lingual Frenulum	116 (19.0)	115 (44.1)	1 (0.3)	< .001	1 (2.9)	1 (3.8)	0 (0.0)	.581	< .001
Restricted Tongue Movement	184 (30.1)	3 (1.1)	181 (51.6)	< .001	0 (0.0)	0 (0.0)	0 (0.0)	-	< .001

Surgical correction

Of those diagnosed with ankyloglossia, surgical treatment was performed in 487 (79.6%) cases. Frenotomy was significantly more likely to be performed in the inpatient hospital setting (p=.018) and in infants in which ankyloglossia was actually diagnosed (p<.001) (Table 6). Painful latch (p<.001) and poor latch (p<.001) were feeding complaints that were found to be significantly associated with patients who received frenotomies. Similarly, notched tip (p=.005), lack of extension beyond the alveolar ridge (p<.001), taut frenulum (p=.036), short lingual frenulum (p<.001), and restricted tongue movement (p<.001) were significantly associated with those who had surgical correction (Table 7). A logistic regression was performed to ascertain the effects of location, age, and various feeding difficulties on the likelihood that frenotomy was performed. The logistic regression model was statistically significant (χ^2^(8) = 152.653, p<.001). The model explained 31.4% (Nagelkerke R^2^) of the variance in frenotomy and correctly classified 80.5% of cases. Ankyloglossia was 4.03 times more likely to be diagnosed in the hospital compared to the clinic. Complaints of painful latch were associated with a 4.24 times increased likelihood of frenotomy, infants with poor latch were 4.44 times more likely to undergo the procedure, and other feeding complaints were seen to have a 2.43 times higher incidence of frenotomy being performed.

**Table 6:** Frenotomy procedure rates by diagnosis and location p-values calculated using the chi-square test

Frenotomy Performed, N (%)	Ankyloglossia Diagnosis				No Ankyloglossia Diagnosis				Total p-value
	Total	Clinic	Hospital	P Value	Total	Clinic	Hospital	p-Value	
Yes	487 (79.6)	196 (75.1)	291 (82.9)	.018	0 (0.0)	0 (0.0)	0 (0.0)	-	< .001
No	125 (20.4)	65 (24.9)	60 (17.1)	-	34 (100)	26 (100)	8 (100)	-	-

**Table 7:** Pre-frenotomy characteristics p-values calculated using the chi-square test

	Frenotomy Done (N=487)	No Frenotomy Done (N=159)	p-value
Feeding Complaints, N (%)			
Painful Latch	289 (59.3)	36 (22.6)	< .001
Poor Latch	323 (66.3)	46 (28.9)	< .001
Prolonged Feeding	18 (3.7)	7 (4.4)	.674
Poor Weight Gain	24 (4.9)	12 (7.5)	.202
Other	57 (11.7)	39 (24.5)	< .001
None	36 (7.4)	45 (28.3)	< .001
Tongue Descriptors, N (%)			
Normal	3 (0.6)	38 (23.9)	< .001
Notched Tip	68 (14.0)	12 (7.5)	.005
No Extension of Tongue Beyond Alveolar Ridge	68 (14.0)	0 (0.0)	< .001
Taut Frenulum	23 (4.7)	16 (10.1)	.036
Short Lingual Frenulum	103 (21.1)	13 (8.2)	< .001
Restricted Tongue Movement	167 (34.3)	17 (10.7)	< .001

## Discussion

The otolaryngology community generally describes ankyloglossia as a “condition of limited tongue mobility caused by a restrictive lingual frenulum” [3]. In recent years, the diagnosis of ankyloglossia has increased; Walsh et al. reported an 834% increase in diagnoses of ankyloglossia between 1997 and 2012, with the greatest increase between 2006 and 2012 [2]. This growth has been partly attributed to speculation that ankyloglossia is a contributing factor in breastfeeding failure, as it has been associated with ineffective latching, decreased ability to create a seal, poor infant weight gain, and maternal nipple pain [3,4]. While some studies have shown that surgical intervention can be beneficial in cases of breastfeeding difficulties, other larger systematic reviews have found inadequate evidence that this is the case [5,6]. Some individuals have also hypothesized that ankyloglossia disturbs speech development. However, many studies have failed to show an association between ankyloglossia and delayed onset of speech or speech disorders [7,8]. A recent consensus statement put out by the American Academy of Otolaryngology-Head and Neck Surgery Foundation (AAO-HNS) speculated that an increasing number of lactation consultants identifying ankyloglossia, increasing social media attention of the topic, and an increasing number of medical practitioners and primarily dentists treating ankyloglossia have also contributed to the rise in the number of children diagnosed with ankyloglossia. The AAO-HNS notes that an additional reason for the increase in incidence is the expanding description of ankyloglossia. To better understand the increasing trend in ankyloglossia incidence, this study sought to understand whether patients were being appropriately referred to otolaryngology for ankyloglossia treatment, and how often those patients were diagnosed with ankyloglossia.

Who is being referred?

Patients referred for ankyloglossia were more often male (62.8% N=406), paralleling previous reports of a male preponderance in children diagnosed with ankyloglossia [9,10]. While ankyloglossia has been reported as a symptom of rare cases of X-linked cleft palate [11], no patients in this study were noted to have a cleft palate. Some studies have attributed the male predisposition to a genetic origin, but no definitive mechanism has been identified [4].

The mean age of patients in the hospital setting was younger than that of the outpatient setting. This can be explained by the fact that many of the inpatient consults were referred by lactation consultants or pediatricians during routine physical exam prior to newborn discharge, as opposed to outpatient consults that likely arose due to feeding difficulties mothers experienced while nursing after leaving the hospital.

Diagnosis

Although there is controversy surrounding ankyloglossia, there is a general consensus that a diagnosis of ankyloglossia consists of limited tongue mobility and/or a restrictive lingual frenulum and is properly evaluated by a careful history and physical examination, including inspection and palpation [3]. Following diagnosis, the reporting of the finding proves to be an area of confusion. While there is a consensus regarding the term “anterior” ankyloglossia referring to a lingual frenulum that extends to the tip of the tongue, “posterior” ankyloglossia has been noted to indicate a frenulum that inserts at a distance from the tongue tip as well as potentially a submucosal tethering of the tongue [3,12]. Of the patients referred for ankyloglossia, 94.7% (N=612) patients were diagnosed with ankyloglossia. Within patient charts, this was reported in a variety of means, including the Coryllos system, the Bristol Tongue Assessment Tool, and the Hazelbacker Assessment Tool for Lingual Frenulum Function, as well as using stand-alone “anterior” and “posterior” terms. Due to the lack of consistent language in describing each diagnosis, it is impossible to conclude whether each diagnosis was made appropriately and how ankyloglossia has been defined. This local evidence of a wide ankyloglossia definition contributing to an increasing incidence of diagnoses of ankyloglossia parallels that of the field.

Feeding complaints

In addition to the widening definition of ankyloglossia, the over-diagnosis of ankyloglossia may be due to the increased attention on the benefits of breastfeeding as well as the increased awareness of the possible negative association between ankyloglossia and breastfeeding [3]. Exclusive breastfeeding for the first six months of life has been shown to benefit the newborn’s immune system and protect the child from disease [13]. Thus, when breastfeeding difficulties occur, a concerned mother may reach out for help. Previously, increasing LATCH scores were observed with decreasing lingual frenulum tethering [14], allowing us to use feeding complaints as a surrogate for a restrictive lingual frenulum.

The most commonly reported feeding difficulties by mothers were poor latch and painful latch; previous reports have also found nipple pain and inadequate latching to be the most common complaints [3,15,16]. In this study, painful latch and poor latch were found to be significantly associated with those who received an ankyloglossia diagnosis as well as those who had frenotomies performed, corroborating the idea that feeding complaints play a large role in the attention on ankyloglossia for mothers. Although painful latch and poor latch are normal at the beginning of breastfeeding, they were more significantly associated with the inpatient hospital setting; this may contribute to the reason for a more significant number of frenotomies being performed in the hospital (p=.018). Amongst those diagnosed with ankyloglossia, complaints of painful latch (p<.001) and poor latch (<.001) were significantly associated with the hospital setting, while complaints of prolonged feeding (p=.014) and poor weight gain (p<.001) were significantly associated with the clinic setting. As clinic patients were on average six weeks older than those seen in the inpatient setting, complaints of prolonged feeding and poor weight gain are most likely more associated with the outpatient setting as these complaints require some time to observe.

Tongue descriptors

 In accordance with the AAO-HNS consensus statement finding of ankyloglossia consisting of limited tongue mobility and/or a restrictive lingual frenulum, the most common tongue descriptor found was restricted tongue movement. Those diagnosed with ankyloglossia and those who received surgical correction were significantly more likely to have abnormal anatomy (Tables 5, 7). This evidence provides support for the fact that the attention on ankyloglossia has a feeding component but also abnormal anatomy, namely restricted movement, is a necessary component. In comparing those diagnosed with ankyloglossia in the clinic versus the hospital, no extension beyond the alveolar ridge, taut frenulum, short lingual frenulum, and restricted tongue movement were found to be significantly different in prevalence between the two settings. This may be due to a preference in reporting by certain physicians in the hospital versus clinic setting.

Frenotomy

Although 94.7% of patients were diagnosed with ankyloglossia, only 79.6% of those patients underwent frenotomy. Possible reasons for the discrepancy between the frequency of diagnoses and the frequency of frenotomy include degree of severity of tongue tie, doubt regarding the benefits of frenotomy, and personal parental preference [17]. Overall, there is a clear association between feeding complaints and abnormal tongue anatomy among those who underwent surgical correction as compared with those who did not. While reports have suggested an association between frenotomies and improved breastfeeding [6,18,19], other studies have suggested limited benefit, especially in mild-moderate cases of tongue-tie [20]. Some reports have even said that surgical correction has more of a placebo role [21]. Further research is necessary to understand whether surgical correction truly is beneficial in combating feeding complaints.

Limitations

This study is limited by its retrospective nature and by the fact that diagnosis was made by individuals without concrete criteria. However, there is not a specific widely accepted method of diagnosing ankyloglossia in infants. Also, feeding issues were based on maternal reports and some infants likely had other issues that became clearer with time.

## Conclusions

Analysis of pediatric patients under the age of one referred to otolaryngology for ankyloglossia at a large academic institution revealed that patients were often being referred for appropriate breastfeeding complaints and were diagnosed with ankyloglossia most of the time. There is still a knowledge gap among primary care providers as a small group was referred in the absence of feeding issues. The big questions about ankyloglossia remain unanswered. These are how to diagnose ankyloglossia accurately and how effective frenotomy is, in the appropriate case, to improve breastfeeding issues in new mothers.
